# Biology of sepsis: Its relevance to pediatric nephrology

**DOI:** 10.1007/s00467-013-2677-3

**Published:** 2014-01-10

**Authors:** Neal B. Blatt, Sushant Srinivasan, Theresa Mottes, Maureen M. Shanley, Thomas P. Shanley

**Affiliations:** 1 Division of Pediatric Nephrology, C.S. Mott Children’s Hospital at the University of Michigan, Ann Arbor, MI USA; 2 Department of Pediatrics, University of Wisconsin School of Medicine and Public Health, Madison, WI USA; 3 Division of Pediatric Nephrology, Cincinnati Children’s Hospital Medical Center, Cincinnati, OH USA; 4 Division of Pediatric Critical Care Medicine, C.S. Mott Children’s Hospital at the University of Michigan, Building 400 2800 Plymouth Road, Ann Arbor, MI 48109 USA

**Keywords:** Sepsis, Multi-organ dysfunction, Intensive care, Acute kidney injury, Host immune response, Systemic inflammatory response syndrome

## Abstract

Because of its multi-organ involvement, the syndrome of sepsis provides clinical challenges to a wide variety of health care providers. While multi-organ dysfunction triggered by sepsis requires general supportive critical care provided by intensivists, the impact of sepsis on renal function and the ability of renal replacement therapies to modulate its biologic consequences provide a significant opportunity for pediatric nephrologists and related care providers to impact outcomes. In this review, we aim to highlight newer areas of understanding of the pathobiology of sepsis with special emphasis on those aspects of particular interest to pediatric nephrology. As such, we aim to: (1) review the definition of sepsis and discuss advances in our mechanistic understanding of sepsis; (2) review current hypotheses regarding sepsis-induced acute kidney injury (AKI) and describe its epidemiology based on evolving definitions of AKI; (3) review the impact of renal failure on the immune system, highlighting the sepsis risk in this cohort and strategies that might minimize this risk; (4) review how renal replacement therapeutic strategies may impact sepsis-induced AKI outcomes. By focusing the review on these specific areas, we have omitted other important areas of the biology of sepsis and additional interactions with renal function from this discussion; however, we have aimed to provide a comprehensive list of references that provide contemporary reviews of these additional areas.

## Defining “sepsis”

While the response of the human host to an invasive pathogen has been recognized for centuries, it was only in the early 1990s that any attempt at developing a consensus definition for sepsis (and septic shock) was made [1]. It was believed that a standardized definition would afford accurate determination of the epidemiology of sepsis and serve to identify patients with sepsis for the purpose of study enrollment in clinical trials. Thus, in 1992, participants of the American College of Chest Physicians and Society of Critical Care Medicine Consensus Conference first derived what remains the basis for the most widely accepted definition for sepsis [1]. Nearly a decade later, an international panel of experts in sepsis refined the adult consensus definition of sepsis (and related terms) and adapted the definitions for children [2]. The definition was based on the clinical observations of tachycardia, tachypnea, hyperthermia (or hypothermia) as well as laboratory evidence of leukocytosis and/or significant bandemia (>10 % bands in a differential white blood cell count) in the setting of proven or highly suspected infection. While these clinical findings reflected the host’s systemic response to infection, it was clear that similar responses could be observed without evidence or involvement of a pathogen trigger (e.g., in response to pancreatitis, trauma, etc.). As a result, the terms “*systemic inflammatory response syndrome*” or *SIRS* was used to characterize this state of immune activation in which an infant or child showed the presence of at least two of these four criteria, one of which must be either hyper/hypothermia or leukocytosis (Table 1). Numerous critical clinical conditions are associated with SIRS criteria, so it has been argued that this definition is not specific enough to provide any meaningful clinical distinction among triggering etiologies—both pathogen and non-pathogen causes alike [3]. Nevertheless, the term “*sepsis*” is applied to those states in which SIRS criteria are present in response to an infection. The infection can be established either by positive microbiology cultures, clear clinical evidence of infection (such as a culture negative empyema) or with strong clinical suspicion. When there is evidence of inadequate end-organ perfusion (see criteria in Table 2) resulting from this systemic response, the term “*severe sepsis*” is applied. For pediatrics, severe sepsis encompasses cardiovascular dysfunction, though many use the specific descriptor of s*eptic shock* to define sepsis associated with hypotension (two distinct measurements < 3rd percentile for age) after the administration of at least 40 ml/kg of crystalloid or colloid and/or other criteria (see Table 2).

**Table 1:** Clinical and laboratory criteria for the systemic inflammatory response syndrome (SIRS). Note: Patients must present with at least two of the following and *must include either 1 or 4

1. *Temperature > 38° C or < 36° C (as determined by central temperature)
2. Heart rate > 90th percentile for age
3. Respiratory rate >90th percentile for age, or hyperventilation to PaCO_2_ < 32 torr
4. *White blood cell count >12,000 cells/mm^3^, or <4,000 cells/mm^3^

**Table 2:** Criteria for severe sepsis. Sepsis plus any one of the following:

1. Cardiovascular dysfunction:
Despite administration of isotonic intravenous fluid bolus of ≥40 ml/kg in 1 h: Decrease in blood pressure to <5th percentile for age or blood pressure at <2 SDs below normal for age; or Need for vasoactive drug to maintain blood pressure in the normal range (dopamine > 5 mcg/kg/min or dobutamine, epinephrine, or norepinephrine at any dose); or
Any two of the following
Unexplained metabolic acidosis (base deficit > 5.0 mEq/l);
Increased arterial lactate at >2 times the upper limit of normal;
Oliguria (urine output < 0.5 ml/kg per h);
Prolonged capillary refill (>5 s); or
Core-to-peripheral temperature differential of > 3 °C
2. Respiratory
PaO_2_/FiO_2_ < 300 in absence of cyanotic heart disease or preexisting lung disease; or
PaCO_2_ > 65 torr or 20 mm Hg over baseline PaCO_2_; or
Proven need or 0.5 FIO_2_ to maintain saturation at ≥92 %; or
Need for non-elective invasive or non-invasive mechanical ventilation
3. Neurologic
Glasgow Coma Score ≤ 11; or
Acute change in mental status with a decrease in Glasgow Coma Score of ≥3 points from abnormal baseline
4. Hematologic
Platelet count < 80,000/μl or a decline of 50 % in platelet count from highest value recorded over the past 3 days (for patients with chronic thrombocytopenia); or
International normalized ratio > 2
5. Renal
Serum creatinine level ≥2 times the upper limit of normal for age or twofold increase in baseline creatinine level
6. Hepatic
Total bilirubin concentration ≥ 4 mg/dl (excepting newborns); or
ALT 2 times upper limit of normal for age

Note: Criteria for septic shock

Sepsis with hypotension (two distinct measurements of blood pressure < 3rd percentile for age) after administration of 40 ml/kg of crystalloid or colloid, plus any one of the following:

1. Requirement for inotropic or vasopressor support (excluding dopamine < 5 μg/kg/min)

2. Any of the cardiovascular diagnostic criteria for severe sepsis listed above

Conventional management of pediatric sepsis/septic shock has been reviewed elsewhere [4, 5], and so it will not be detailed here. Current guidelines place an emphasis on early fluid resuscitation with isotonic fluids up to 60 ml/kg. If on-going hypoperfusion or frank hypotension is still observed, the patient is described to have ‘fluid-refractory shock’ and initiation of inotropic support (either dopamine or low-dose [0.02–0.1 mcg/kg/min] epinephrine) is recommended. If inadequate end-organ perfusion is still evident, the patient is described as having ‘catecholamine-refractory shock’ and it behooves the clinician to determine whether the patient is demonstrating ‘cold’ shock or ‘warm’ shock. Cold shock is manifested by decreased perfusion characterized by delayed capillary refill (>3 s), and cool, mottled extremities and is associated with low cardiac output and elevated systemic vascular resistance—a common hemodynamic pattern in children [6, 7]. ‘Warm shock,’ most commonly observed in adults, is characterized by flash capillary refill, bounding peripheral pulses and a wide pulse pressure and is associated with high to supranormal cardiac output with significantly reduced systemic vascular resistance. It is important to recognize that these hemodynamic states of fluid-refractory, catecholamine-resistant shock can vary over time such that real-time, bedside attention to the clinical hemodynamic pattern is necessary. This recommended algorithmic approach to managing pediatric shock is currently undergoing its third iteration with plans to be published in 2014 [4].

## Pathogenesis of sepsis

The ability to contain and eradicate invading pathogens has enabled humans to evolve in a climate of numerous microbial challenges. This fundamental function necessary for host survival is served by the immune system that has elegantly evolved to contain and eradicate pathogens while preserving physiologic integrity of the host. In the latter half of the 20th century, combined clinical and basic science studies demonstrated that pathogens (and/or their products) were responsible for initiating a host immune response that resulted in SIRS. While this response is typically well regulated by the host, dysregulation of this local response can lead to tissue injury and consequent organ dysfunction. Pediatricians are well aware of the various inciting pathogens that can trigger this physiologic response: Gram-negative and Gram-positive bacteria, viruses, fungi, and even protozoa. Failure to contain and/or eradicate microbes leads to overwhelming, systemic spread of pathogens (e.g., bacteremia) with subsequent release of toxins (e.g., endo- or exotoxins) that can directly injure cells resulting in organ dysfunction. Thus, higher-order organisms deploy a robust immune system comprised of two separate arms: the innate immune system that provides relatively immediate and non-specific immunity and the adaptive immune system that provides the system’s “memory” arm. While this dichotomization is simplistic conceptually, in fact the two arms are integrally linked.

The innate immune system is responsible for initial pathogen recognition and subsequent mounting of an effector response. To fulfill the responsibility of surveying the host environment for foreign pathogens, cells of the innate immune system (e.g., monocytes, macrophages, neutrophils, natural killer [NK] cells) have acquired a series of cell-surface receptors called *pattern-recognition receptors* (*PRRs*) that detect molecular patterns foreign to the host [8]. These receptors are capable of recognizing conserved molecular moieties expressed by various pathogens termed “microbial- or pathogen-associated molecular patterns” (MAMPs or PAMPs) that include various components of invading pathogens, such as lipopolysaccharide (LPS) from Gram-negative bacteria. Among the most important family of PRR’s are the Toll-like receptors (TLR) that possess specificity for the variety of pathogens that infect humans (Table 3) (reviewed in [8]). In addition to the TLRs, other intracellular PRRs exist including nucleotide-binding oligomerization domain (NOD)-like receptors (NLRs) that detect peptidoglycan of Gram-positive bacteria in the cytosol and retinoic-acid-inducible protein (RIG)-like receptors (RLRs) that detect viral double-stranded RNA to induce type I interferon production [9]. Engagement of PRR’s by MAMPs triggers an effector response mediated by consequent activation of cells of the innate immune system that drives expression of numerous inflammatory mediators. It is worth noting that while pathogens trigger immune activation, it is well understood that endogenous molecules released upon tissue damage and/or cellular disruption (e.g., high mobility group protein B1 [HMGB1], S100 proteins, and heat shock proteins) are similarly capable of activating the immune system through PRRs resulting in a systemic response indistinguishable from sepsis (reviewed in [10]). Such molecules are referred to as “damage-associated molecular patterns” (so-called DAMPs) or *alarmins* that can trigger systemic release of immune mediators [11]. Regardless of the trigger, these circulating mediators are responsible for activation and recruitment of cells targeting localized pathogen killing; however, when their expression is dysregulated, systemic spillover can affect numerous physiologic perturbations that are the hallmark of sepsis.

**Table 3:** Pathogen/microbial-associated molecular patterns (P/MAMP) and corresponding human Toll-like receptors (TLRs) and microbial source (pathogen)

P/MAMP	PRR	Pathogen
Peptidoglycan, Pam3CSK4^a^, Zymosan	TLR1 TLR2 TLR6^b^	Gram-positive bacteria
dsRNA	TLR3	Virus
LPS, Lipid A	TLR4	Gram-negative bacteria
Flagellin	TLR5	Bacteria, Flagellum
ssRNA	TLR7 TLR8	Virus
CpG DNA motifs	TLR9	Bacteria, DNA
Profilin-like proteins	TLR10	

PRR, pattern recognition receptors

^a^Synthetic triacylated lipopeptide

^b^Heterodimerizes with TLR1, TLR2

One of the earliest identified cytokines causally linked to sepsis is tumor necrosis factor-α (TNF). TNF drives adhesion molecule and chemokine expression to facilitate leukocyte-endothelial cell adhesion and cause endothelial barrier dysfunction (reviewed in [12]). TNF upregulates tissue factor and inhibits protein C to contribute to a pathologic pro-coagulant state within the microvasculature and also mediates pathologic vasodilation, myocardial depression and afferent renal artery vasodilation by inducing nitric oxide (NO) synthase and NO production to potentially reduce transglomerular pressure [13]. A related cytokine, interleukin (IL)-1β elicits fever, hypotension and leukocytic infiltration to numerous organs in animal models. IL-1β also activates monocytes and increases adhesion molecule expression as well as tissue factor expression while inhibiting thrombomodulin secretion. These effects contribute to a procoagulant state in sepsis that leads to disseminated microvascular thrombosis and thus, end-organ ischemia—including within the kidney. There are several other important proinflammatory cytokines and mediators that contribute to sepsis pathology that are beyond the scope of this review [14, 15]. It is important to recognize that while inflammation is necessary for pathogen clearance, this immune response must ultimately be contained in a controlled manner in order to reestablish homeostasis and avoid cellular and tissue damage. This function is served by a counter-regulatory immune response.

To maintain immune homeostasis following infection, the proinflammatory response must be down-regulated. This function is mediated by a counter-regulatory, anti-inflammatory response ‘syndrome’ (so-called “CARS”) that results from expression of various endogenous cytokine antagonists, including soluble TNF receptors that bind to and inactivate TNF, interleukin-1 receptor antagonist (IL-1Ra) that blocks IL-1 effect and other anti-inflammatory cytokines, such as IL-10 and transforming growth factor (TGF)-β. IL-10 directly inhibits expression of cytokines known to contribute to sepsis and also increases expression of other anti-inflammatory molecules such as IL-1Ra and soluble TNF receptors (reviewed in [16]). Importantly, it has become clear that just as with the pro-inflammatory response, the compensatory response can also be dysregulated [17]. When prolonged, this dysregulation may impair pathogen clearance via over-suppression of the immune response and impact mortality from sepsis. Data from gene expression profiling of children dying from septic shock have demonstrated significant repression of genes associated with critical immune functions: antigen presentation, T-cell and B-cell activation and natural killer cell function [18]. In addition to reduced gene expression, functional defects in several immune cell types characterized by impaired activation of monocytes, T-cells and neutrophils by pathogens have been described in the later phase of sepsis [19, 20]. Post-mortem analysis has found that nearly 80 % of septic adults had evidence of unresolved infection [21], and the common occurrence of nosocomial sepsis, often associated with less virulent, opportunistic infections as well as the reactivation of latent viruses all imply immune dysfunction in the septic host. Given these observations, it is increasingly suggested that monitoring immune function and appropriate, targeted immune adjuvant therapies to enhance immune cell function may hold much more significant promise as a therapeutic approach [22]. As early resuscitative efforts have improved the survival from acute sepsis, it is argued that this ‘late’ phenomenon is responsible for the trend towards later deaths among both adults and children succumbing to sepsis. Given the risks related to immune dysfunction in the patient with sepsis, strict attention to protocols that reduce the occurrence of nosocomial infections (e.g., catheter-associated blood stream infections and ventilator-associated pneumonia) is imperative, as preventing a sepsis trigger may be more important, and achievable, than eradicating an infection once established (see *Sepsis prevention strategies* below).

## Insights as to how sepsis induces acute kidney injury (AKI)

Within the context of sepsis pathology, a primary question for the pediatric nephrologist is how sepsis induces AKI. The logical presumption is that impaired hemodynamics and hypotension lead to an under-perfused state of the kidneys with consequent injury. However, both animal and human studies in sepsis have indicated that maintaining systemic pressure does not necessarily prevent AKI, and that decreases in renal blood flow and glomerular filtration rate can be seen following injections of LPS even when there is no change in systemic blood pressure [23, 24]. This has suggested that the degree of organ dysfunction in humans with sepsis correlates with alterations in microvascular circulation [25–27]. In order to further elucidate mechanistic causes of sepsis-induced AKI, investigators have utilized a variety of experimental models ranging from small to large animal models.

While not necessarily replicating all the physiologic manifestations of human sepsis, murine models using pathogen-associated challenges (e.g., LPS) do produce AKI and have been used to study the role of MAMP-induced inflammatory responses in causing renal injury [28]. Consistent with the cascade described above, this work linked the generation of LPS-induced cytokines (e.g., TNF) to consequent induction of nitric oxide (NO) synthesis [29]. Generation of NO leads to increased reactive nitrogen species and activation of caspases known to contribute to programmed cell death, or apoptosis [30]. Studies have further confirmed the role of reactive nitrogen species and apoptosis in mouse models of sepsis-induced AKI. For example, Lee et al. observed increased renal tubular cell apoptosis in a mouse model of sepsis, and found that inhibition of caspase-3 (a critical mediator of apoptosis) preserved kidney function [31]. Mayeux and colleagues have similarly shown that agents that scavenge or inhibit reactive oxygen and reactive nitrogen species rescue renal peritubular capillary blood flow and decrease renal tubular cell injury [32, 33]. This work concluded that cytokine-mediated endothelial injury and capillary dysfunction along with vasodilation are important factors in the development of AKI [28–30]. Interestingly, in the setting of systemic vasodilation in sepsis, activation of the renin–angiotensin–vasopressin system may cause paradoxical renal vasoconstriction [34], stressing the importance of using alternative models to obtain a better understanding as to how endothelial stress in sepsis impacts peritubular capillary dysfunction that may reveal novel therapeutic approaches [34].

Because several limitations exist in the translation of findings from murine models to humans, and vasculature cannot readily be cannulated in mice, researchers have employed large animal models involving intravenous infusion of live pathogens (e.g., *Pseudomonas aeruginosa*, *E*. *coli*) or autologous fecal peritonitis, in order to discern the physiologic renal responses associated with the consequent development of AKI [35, 36]. These models have demonstrated similar variability in the incidence of sepsis-induced AKI (40–62 %) as is typically observed in older adult cohorts (∼50 % incidence), suggesting they may reasonably reflect human pathophysiology. Benes et al. recently observed that despite being subjected to similar degrees of pathogen challenge, showing comparative hemodynamic responses and receiving uniform fluid and vasoactive support, the only animals (pigs) that developed AKI were those that demonstrated progressive increases in renal vascular resistance which did not correlate with systemic hemodynamics [35]. In fact, consistent with the hypothesized role of inflammation driving renal injury, those animals developing AKI demonstrated an earlier and more robust inflammatory response (measured by circulating TNF-α and IL-6 levels) and higher oxidative stress (measured by thiobarbituric acid reactive species [TBARS]) [35]. Such studies highlight the likely failure of focusing only on management of systemic hemodynamics to ameliorate AKI, however, in the absence of advances in monitoring the capability of organ-specific perfusion, the bedside clinician is left with this ‘crude’ measurement.

Despite the insight gained from these studies, additional questions were raised as a result of observations from a sheep model of sepsis-induced AKI [36]. In this model, infusion of *E*. *coli* over a 48-h interval, followed by antibiotic administration and recovery (for an additional 48 h) induced a hyperdynamic, vasodilatory model of sepsis. These investigators observed renal vasodilation and subsequent increased renal blood flow in the early sepsis phase, but relative renal vasoconstriction and reduction in renal blood flow as the animal recovered, providing further evidence for the role of renovascular resistance (RVR) in inducing AKI and demonstrating the causative mechanism to be much more complex than hypoperfusion due to decreased cardiac output and hypotension. Sepsis-induced renal microvascular alterations triggered by vasoconstriction, capillary leak with consequent tissue edema, endothelial dysfunction with microthrombosis (see below), and/or elevated intra-abdominal pressure might all contribute to increased RVR. This may also explain the increased incidence of AKI in adults with sepsis who require systemic vasopressors (e.g., norepinephrine and/or vasopressin) to maintain adequate systemic blood pressure.

With all these models it remained incompletely understood as to whether systemic and/or local (kidney produced) cytokines were predominantly responsible for triggering AKI. ‘Traditional’ thinking had surmised that TLR-dependent activation of circulating leukocytes led to their activation and consequent systemic cytokine production that either directly or indirectly contributed to renal injury. However, mammalian renal tubular cells constitutively express a number of TLRs (1, -2, -3, -4, and −6) [37] and in vitro stimulation with LPS can increase their expression of both inflammatory mediators, as well as TLR’s (2, 3, and 4) themselves [38]. These data raise the possibility that tubular cell TLR’s might locally direct the recruitment of interstitial leukocytes into the kidney with consequent tubular injury during systemic sepsis (reviewed in [38]). Delineating this was testable by an elegant study of LPS-induced AKI utilizing transgenic mice deficient in TLR4 expression. These mice, which lack a systemic TNF response to LPS, were found to be resistant to endotoxin-induced AKI [28]. However, using cross transplantation, these investigators showed that while TLR4-deficient recipients of wild-type kidneys developed minimal LPS-induced AKI, wild-type recipients of a TLR4-deficient kidney did in fact develop severe acute renal injury after endotoxin [28]. Thus, these data implicate the systemic inflammatory response during sepsis as the primary driver of AKI (and very likely other organs) in sepsis. Interestingly, converse findings were obtained when the specific role of TNF was examined using TNF receptor-1 (TNFR1)-deficient mice [29]. In these studies, TNFR1 null mice were resistant to LPS-induced AKI with less renal inflammation and reduced apoptosis of tubular cells providing support for a role of cytokines in causing tubular cell apoptosis as a putative mechanism for sepsis-induced AKI [39, 40]. Furthermore, in contrast to TLR4 null mice, TNF receptor (TNFR)-positive kidneys that were transplanted into TNFR null mice did show evidence of local injury and AKI, whereas the TNFR null kidneys transplanted into wild type mice did not. These data suggest that TNF plays a very direct and local role in LPS-triggered AKI [29], though exactly how remains incompletely known.

Though there is ample evidence that systemic inflammation in sepsis is a key driver of end-organ dysfunction, there is an inconsistent association of AKI with sepsis-induced alterations in systemic hemodynamics, which suggests other mechanisms may be at play. As mentioned earlier, the “cytokine storm” of proinflammation concomitantly activates the coagulation cascade and impairs fibrinolysis, driving a functional hypercoagulable state that can ultimately lead to disseminated microvascular thrombosis [41]. An early link between inflammation and activation of the coagulation cascade in contributing to organ dysfunction was supported by autopsy findings of clots comprised of neutrophils, platelets and fibrin in the microcirculation of patients dying of sepsis-induced multiple organ failure likely as a result of ischemia [42]. This pathophysiology is often characterized by platelet consumption with subsequent thrombocytopenia so that clinician investigators refer to this cause of multiple organ dysfunction as thrombocytopenia-associated multiple organ failure (TAMOF) [43]. TAMOF is characterized as a thrombotic microangiopathic process, similar to that described in thrombotic thrombocytopenic purpura (TTP), which is characterized by decreased ADAMTS13 protease activity causing accumulation of large, multimeric von Willebrand Factor (vWF) [44]. As a pro-thrombogenic protein released into the circulation by the vascular endothelium and platelets, vWF provides hemostatic function at sites of vascular injury [45]. In the normal state, circulating proteases, notably ADAMTS13, degrade vWF into smaller multimers thereby reducing the thrombogenic propensity of vWF; however, in sepsis this homeostatic function may go awry. For example, in some cases of severe sepsis, attenuation in the release of ADAMTS13 results in the formation of large vWF multimers causing disseminated microvascular thrombosis and organ failure [45]. This observation would suggest that plasma exchange, by clearing large vWF multimers and replacing ADAMTS13 with fresh plasma, may improve organ dysfunction/AKI by diminishing circulating vWF multimers. These results have important implications for the use of extracorporeal therapies, notably continuous renal replacement therapy combined with plasmapheresis/exchange in treating sepsis and sepsis-induced AKI—particularly in the specific setting of TAMOF.

In summary, sepsis-induced AKI appears to be driven by release of systemic, rather than local, inflammatory cytokines. However, the contribution of systemic hemodynamic changes appears less important than local changes in renal perfusion mediated by imbalance of afferent and efferent vasodilation/contraction—the cause(s) of which remain to be fully elucidated. Tubular cell apoptosis and microvascular thrombosis, both driven by systemic release of proinflammatory cytokines, may also be important contributing factors. It remains imperative to better understand this multifactorial pathology since the health care burden of sepsis-induced AKI as reflected by the epidemiology is substantial.

## Epidemiology of sepsis-induced AKI

Sepsis is a common reason for admission to the pediatric intensive care unit (ICU) and carries significant mortality, morbidity, and cost to the health care system [2]. In the past, epidemiological studies of pediatric sepsis were hampered by non-standard definitions and a similar deficiency has impaired accurate reporting of the incidence of pediatric AKI. Noting over 30 definitions of AKI in published literature, the Acute Dialysis Quality Initiative Group proposed the Risk, Injury, Failure, Loss, End-Stage Kidney Disease (RIFLE) system to standardize definitions (Table 4) [46]. These criteria were modified for pediatrics to yield the pRIFLE score that has been proposed for prospective studies examining AKI in children [47]. Further modifications of RIFLE are reflected in the Acute Kidney International Network scoring system and in the Kidney Disease Improving Global Outcomes (KDIGO) clinical practice guideline for AKI, though these have yet to be validated in pediatrics [48, 49]. Early studies were confounded by non-standard definitions for sepsis, AKI, or both; nevertheless, these historical data provide general insight into the burden and impact from sepsis-induced AKI. With the establishment of a consistent scoring system capable of better defining the incidence of AKI in children, studies clearly show that sepsis is not only one of the most common etiologies for AKI, but that the combination of AKI and sepsis results in worse outcomes than either entity alone.

**Table 4:** Acute kidney injury classification systems

System	Injury stage	Criteria (serum creatinine)	Criteria (urine output)
RIFLE	R (risk)	≥1.5-fold Cr increase or GFR decrease ≥ 25 %	<0.5 ml/kg/h for 6 h
	I (injury)	≥2-fold Cr increase or GFR decrease ≥ 50 %	<0.5 ml/kg/h for 12 h
	F (failure)	≥3-fold Cr increase or Cr >4.0 mg/dl	<0.3 ml/kg/h or anuria for 12 h
	L (loss)	Persistent failure >4 wk	
	E (end stage)	Persistent failure >3 mo	
pRIFLE	R (risk)	GFR decrease ≥ 25 %	<0.5 ml/kg/h for 6 h
	I (injury)	GFR decrease ≥ 50 %	<0.5 ml/kg/h for 12 h
	F (failure)	GFR decrease ≥ 75 % or eGFR < 35 ml/min/1.73 m^2^	<0.3 ml/kg/h or anuria for 12 h
	L (loss)	Persistent failure >4 wk	
	E (end stage)	Persistent failure >3 mo	
AKIN	Stage 1	≥ 0.3 mg/dl Cr increase or 150–200 % increase above baseline	<0.5 ml/kg/h for 6 h
	Stage 2	Cr increase of 200–300 % of baseline	<0.5 ml/kg/h for 12 h
	Stage 3	Cr increase ≥ 300 % of baseline or ≥4.0 mg/dl with an acute increase of 0.5 mg/dl	<0.3 ml/kg/h or anuria for 12 h
KDIGO	Stage 1	≥ 0.3 mg/dl Cr increase or 1.5–1.9 times baseline	<0.5 ml/kg/h for 6–12 h
	Stage 2	Cr increase of 2.0–2.9 times baseline	<0.5 ml/kg/h for ≥12 h
	Stage 3	Cr increase of 3.0 times baseline or Cr ≥4.0 mg/dl or initiation of renal replacement therapy or eGFR <35 ml/min per 1.73 m^2^ in patients <18 years	<0.3 ml/kg/h for ≥24 h or anuria for ≥12 h

AKIN, Acute Kidney Injury Network; KDIGO, Kidney Disease Improving Global Outcome; eGFR, estimated glomerular filtration rate

## Early studies not using standardized AKI definitions

In a retrospective review of case records of hospitalized children over 22 years in a university hospital in Thailand, sepsis was noted to be among the major causes of AKI (21.4 %) [50]. Mortality associated with AKI from all causes was 41.5 %, while mortality in those with sepsis-induced AKI was 66.2 % with an odds ratio for mortality of >17 if sepsis was the cause of AKI [50]. In a retrospective, single-center study in Lithuania of children diagnosed with AKI, sepsis was the primary cause in 21.8 % of patients from 1998–2008 [51]. In this report, AKI was defined as urine output <1 cc/kg/h for 6 h, rise of serum creatinine by 26.4 μmol/l (0.3 mg/dl), or an increase in creatinine to >150–200 % of prior baseline. In a retrospective review of children in septic shock, Plotz et al. reported mortality rates were significantly higher in patients with AKI (defined as need for RRT) (57.1 %) versus those without AKI (6.7 %) [52]. Subjects were defined as having septic shock if they remained hypotensive despite fluid resuscitation and dopamine and required any additional vasopressor agent (most commonly norepinephrine). These epidemiologic findings (sepsis triggering ∼1/4 of AKI) and mortality rates from sepsis and AKI being in the range of 30–40 %, have been described in a variety of other retrospective studies [53–55]. In the only prospective study performed before the era of more precise definitions, sepsis was identified as an important (9.1 %) but not the major cause of AKI observed in children admitted to the PICU with other etiologies including hemolytic-uremic syndrome (18.2 %), hematology-oncology disease (18.2 %), and post-cardiac surgery (11.4 %) [56]. Mortality in those with AKI was 27.3 % versus 2.4 % in those who did not have AKI, though the impact of sepsis on mortality in this cohort was not specifically reported. Taken together, these studies clearly establish sepsis is an important and, as suggested below, increasing cause of AKI.

## Studies using standard AKI definitions

While perhaps still controversial insofar as their prognostic value, several recent standardized definitions of AKI have been derived and described for use in children: pRIFLE, the Acute Kidney Injury Network (AKIN) Staging Criteria, and the KDIGO clinical practice guideline for AKI (see Table 4) [47–49]. The publication and use of these definitions has enabled a more accurate delineation of the association of sepsis with the development of AKI. AKI in hospitalized children using both standard definitions (for sepsis and AKI) has consistently been associated with poor short-term outcomes (notably, mortality and length of stay) [2, 47, 48, 50]. A retrospective, single-center study of PICU patients requiring mechanical ventilation for >4 days utilized the pRIFLE criteria for identifying the incidence of AKI in this cohort [57]. Sepsis was significantly more prevalent in those with AKI (20 %) versus those without AKI (5 %) and mortality was five-times higher in those with AKI (25 %) versus those without (5 %). In the initial validation study of pRIFLE, 150 children admitted to the PICU were prospectively enrolled over a 12-month period and 82 % developed AKI as determined using pRIFLE criteria [47]. The remaining 18 % served as controls for comparison. Between these two cohorts, there were no significant differences insofar as PICU admission diagnoses, including SIRS/sepsis/septic shock (18.5 % in control group, 26.8 % in AKI group;* p* value >0.05). However, these data were collected on a general PICU population, and studies in more severely ill cohorts amplify the incidence and impact of AKI in sepsis.

For example, a retrospective study of burn patients in the PICU applied the pRIFLE criteria and found the incidence of AKI to be 45.5 % and those with AKI developed sepsis during their PICU stay more frequently than those without (38.4 versus 19.8 %) [58]. In addition, 82 % of patients who developed AKI late (after 5 post-burn days) had documented episodes of sepsis as compared to only 19 % of those who had earlier occurrence of AKI presumed to be related to acute, burn-related hemodynamic compromise. A prospective multicenter study in Turkey used standardized definitions for AKI (pRIFLE) and sepsis to identify the association of AKI with sepsis [59]. Sepsis was the cause of AKI in 18.2 % of patients, exceeded only by hypoxic-ischemic injury (28 %). Septic shock was present in nearly a quarter of the children with sepsis and multivariate logistic regression analysis indicated that the presence of septic shock and AKI predicted mortality in patients >1 month old [59]. Overall, there has been a paucity of similar large, cohort studies that have combined the use of standard definitions for both sepsis and AKI, thus the true incidence and interaction of sepsis and AKI remains incompletely defined in pediatrics. Nevertheless, these few studies indicate that the presence of sepsis is a risk factor for development of severe AKI and a significant predictor of mortality.

While many studies associate sepsis with AKI, causality has been much more difficult to prove. Multiple factors (including sepsis) may result in AKI, and differentiating the contribution of any one insult is challenging. Therefore, the predictive capability of renal-specific scores may be insufficient such that an alternative approach that incorporates confounding factors has been proposed [60]. Basu et al. suggested such a methodology to improve prediction of AKI using a concept described as “renal angina” to enhance AKI biomarker efficiency [60, 61]. This proposed clinical guideline integrates baseline, contextual and clinical evidence of kidney injury such that the “risk of AKI” multiplied by the “evidence of AKI” provides a so-called *renal angina threshold*—much like the presence of myocardial infarction risk factors in the presence of chest pain triggers a troponin determination. These investigators provided a diagnostic framework on the basis of risk stratifications by factoring the clinical state and evidence of renal dysfunction such that as the clinical risks increase, the need for evidence of renal dysfunction decreases (see Table 5) [61]. This approach was recently tested in a multi-center retrospective cohort and found to be superior to existing AKI classification systems based simply on serum creatinine and urine output [62]. Whether this approach can be applied in future prospective studies to better predict sepsis-induced AKI and its impact in children remains to be seen. However, such efforts may help earlier identification of optimal therapies to ameliorate the impact of sepsis on AKI and overall clinical outcomes.

**Table 5:** Pediatric renal angina criteria*

Risk category	Renal angina threshold
Moderate risk	
• PICU patient	• Doubling of SCr, *OR* • eCrCl decrease >50 %, OR • Fluid overload >15 %
High risk	
• Acute, decompensated heart failure	• Serum Cr increase >0.3 mg/dl, OR • eCrCl decrease 25–50 %, • Fluid overload >10 %
• Stem-cell transplant	
Very high risk	
• Receiving mechanical ventilation and one or more vasoactive medications	• Any serum Cr increase • eCrCl decrease >25 %, • Fluid overload >5 %

PICU, Pediatric Intensive Care Unit

*Adapted from references [61, 62], used with permission

## Impact of kidney injury/failure on sepsis risk

To this point, we have focused on the epidemiology and pathophysiology of sepsis and its effect on causing AKI. However, it is imperative for the pediatric nephrology community to recognize the potential impact of AKI (and chronic kidney injury/failure) on predisposing the host to sepsis. Very few studies have examined the incidence and potential mechanisms associated with the development of sepsis after established AKI in children. In the largest observational study to date 618 adult ICU patients with AKI (*n* = 391; 64 % of whom required dialysis) were prospectively followed within the five-center Program to Improve Care in Acute Renal Disease (PICARD) [63]. As one would expect, sepsis preceded the onset of AKI in a large number (*n* = 174; 28 %); however, 243 patients (40 %) developed sepsis a median of 5 days after the onset of AKI. Interestingly, this percentage was similar to that reported from a smaller, single-center study of contrast-induced AKI in which 45 % of patients (21/47) developed sepsis after AKI [64]. One would speculate that the invasive nature of central venous or peritoneal catheters and institution of dialytic therapies would predispose this cohort to developing sepsis post-AKI and in fact, the need for dialysis was identified as an independent risk factor [64]. Other independent risk factors for the development of sepsis post-AKI included oliguria ≥3 days, higher percentage of days with fluid overload post-AKI, invasive non-surgical procedures post-AKI and high severity of illness (SOFA) scores. Such observations raise the need for further understanding the potential mechanisms by which renal dysfunction impacts the immune system.

The concept that AKI may impact the immune system to consequently predispose the host to infectious complications has been suggested by the observation that infections are among the most common causes of death in patients with renal failure [65]. This suggests that an unidentified component of renal injury/failure (e.g., uremia) contributes to an immunocompromised state; however, given that severe sepsis is associated with a late phase of immune dysfunction, it is difficult to know which pathophysiologic process may drive this alteration. In trying to identify relevant mechanisms, more work has been done in patients with end-stage renal disease (ESRD) who show a decreased ability to clear bacterial, viral and fungal infections, and show impaired responses to vaccines (e.g., Hepatitis B) and decreased delayed-type hypersensitivity (DTH) (both of which require T-cell-dependent antigen responses) [66]. This may be relevant to sepsis biology as a similar finding of repressed gene expression in T-cell signaling was observed in pediatric septic shock [67]. In contrast to T-cell function, patients with ESRD appear to have intact B-cell function with normal levels of total immunoglobulins (in the absence of proteinuria) and intact vaccine responses to T-cell-independent polysaccharide antigens [66, 68].

Investigators have tried to identify the cellular immune alterations that result from renal dysfunction/failure; however, the results are inconsistent. This is likely related to small cohort sizes, heterogeneity of precipitating causes of the renal failure and variable host genetics. Also, the majority of the samples have been obtained from patients already on dialysis, making it difficult, if not impossible to distinguish those effects resulting from uremia versus the dialysis modality itself. In spite of these confounders, multiple alterations to neutrophil function have been described including decreased chemotaxis, impaired phagocytosis, decreased oxidative burst, and decreased intracellular killing. However, it is unclear how many of these defects were related to the now historical use of bioincompatible cuprophane membranes [69], as recent studies suggest neutrophil phagocytosis and reactive oxygen species production may in fact be preserved in renal failure [70]. With regards to circulating monocytes, key drivers of the sepsis response, numerous studies on adult dialysis patients have demonstrated monocyte activation including upregulation of integrin expression and increased pro-inflammatory cytokine production [71]. Based on these reports, it is widely believed that uremia’s affect on monocytes may contribute to a chronic inflammatory state with increased oxidative stress that in turn contributes to the increased cardiovascular disease in ESRD [72].

In addition to alterations in the cells of the innate immune system, studies of lymphocytes (adaptive immune system) isolated from ESRD patients have shown increased markers of basal activation and apoptosis, but decreased proliferative responses to mitogens [73, 74]. This altered response is similar to lymphocyte dysfunction that has been described in the later phase of pediatric sepsis which is associated with increased risk of both infection and mortality [75]. Additional studies have looked at the impact of ESRD on CD4^+^ T-cell differentiation into T_H_1 and T_H_2 subtypes, as the balance between these subtypes impacts the ability of the immune system to combat infectious challenges. Data regarding the impact of uremia on T-cell function comes from examination of intracellular cytokine staining of freshly isolated CD4^+^ T-cells from ESRD patients. These studies show that uremic patients not on dialysis have increased IL-4- and IL-10-positive T-cells, suggesting a shift towards the immune modulating, T_H_2 phenotype [76]. Given the inherent limitations of studies of circulating leukocytes, it is clear that there is still much to discover about how renal failure and uremia impact immune function, which remains incompletely understood. Given the predilection for developing infections in the chronic renal failure cohort, it is important to adopt management strategies that prevent infections so that sepsis is never established in this at-risk group. To that end, centers should employ strategies to reduce infectious complications (e.g., catheter-associated blood stream infections) in cohorts requiring acute and chronic dialysis.

## Sepsis prevention strategies

**Fig. 1:**
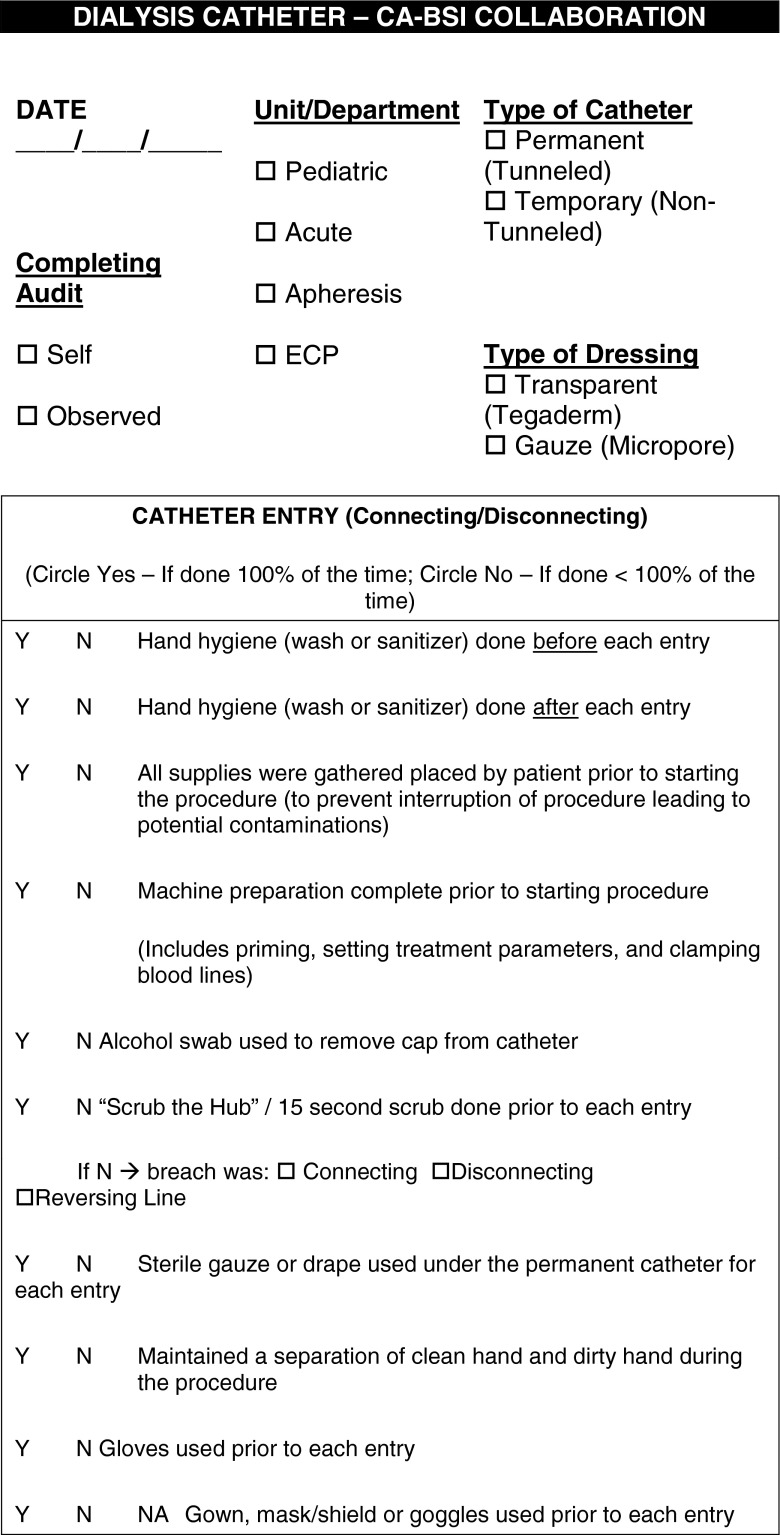

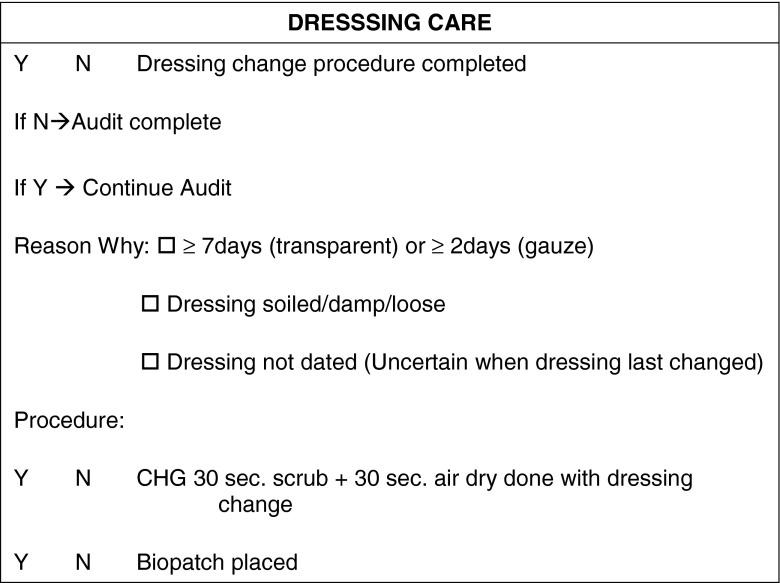
Dialysis catheter CA-BSI checklist. *One sheet for each patient treated on the day of the audit*. Completed forms go in CA-BSI envelope at nurses’ station. Please share comments/suggestions/questions on the back of this form

Recognizing the increased sepsis risk in the cohort of patients with either ESRD or, as noted above, with AKI, our center established a dialysis-specific catheter associated-blood stream infection (CA-BSI) eradication initiative. A multidisciplinary team of nephrologists, dialysis/apheresis nurses, dialysis nurse manager, infection control practitioner, a physician assistant specializing in dialysis catheter insertions, and hospital administration reviewed current best practices for accessing and maintaining dialysis catheters, including recommendations from the Centers for Disease Control (CDC), Centers for Medicare and Medicaid Services (CMS), and the National Association of Children’s Hospitals and Related Institutions (NACHRI; now CHA). A standard procedure for accessing dialysis catheters and maintaining the catheter and its exit site was developed utilizing evidence-based, best practices. An education module demonstrating the accessing procedure was developed by the team who then performed audits to identify breaches in the standardized practice. The information garnered from these audits was used to develop a final checklist of ten critical steps to incorporate into a maintenance bundle focusing on practices that needed to be done at every instance of catheter accessing and exit site dressing changes (Fig. 1). Patients and families also received education from the dialysis/apheresis nurses regarding the updated procedure for accessing and maintaining dialysis catheters.

After implementation of the checklist, compliance was audited over the next 18 months such that each pediatric dialysis patient with a catheter had both self- and anonymously observed audits completed at each encounter and results were reviewed monthly by the eradication team and unit nurses. Areas with low compliance were addressed with staff to identify operational causes for non-compliance and to redesign workflow. In striving toward a 100 % compliance rate, a prominent, visual charting of the metric “Days since last CA-BSI” was displayed in the unit. Most importantly, over time higher compliance rates translated to a statistically significant decrease in the number of CA-BSI’s. In the event a CA-BSI occurred, root cause analysis was performed to identify potential barriers to bundle compliance, disseminate lessons learned and refine preventable measures. Given the challenges inherent to managing children with sepsis, this approach and other related efforts (e.g., antimicrobial lock solutions) aimed at preventing sepsis events by eliminating common triggers are not trivial achievements [77, 78]. Indeed, the KDIGO group’s AKI guidelines devote a chapter on vascular access for RRT in AKI with special attention towards decreasing the rate of catheter-related infections [79].

## Use of renal replacement therapy to modulate the inflammatory response

While prevention of sepsis remains an ideal goal, sepsis will remain a major cause of morbidity and mortality in pediatrics. Due to vascular “leak” from endothelial barrier dysfunction, possibly contributing to and/or in combination with AKI [80], it is common that patients with sepsis develop a fluid-overload state that has been suggested to impact this mortality [81, 82]. As a result, there is a frequent indication for instituting renal replacement therapy to aid in the management of oliguria and consequent volume overload, as well as metabolic acidosis, uremia (notably in the surgical patient where platelet dysfunction/bleeding is an important consideration), maintenance of electrolyte homeostasis and the ability to provide nutrition. Given the typical tenuous hemodynamics of patients with sepsis, continuous renal replacement therapy (CRRT), most commonly in the form of continuous venovenous hemofiltration (CVVH) +/− hemodialysis (HD), is the standard modality used. Perhaps the most controversial decision of applying CRRT is the timing of its initiation. Herein, we do not review the data regarding the impact of fluid overload and possible benefits of earlier initiation of RRT as this has been reviewed in a number of recent reports [83, 84]. Instead, we highlight potential theoretical advantages to utilizing RRT to address the hypothesis that CRRT may provide a beneficial effect on modulation of the immune system in sepsis-induced AKI.

Since the introduction of CRRT into ICU practice, physicians involved in managing AKI patients have considered a paradigm whereby hemofiltration may clear the blood of “bad” pro-inflammatory mediators. First, it is important to acknowledge that it is inaccurate to label any given mediator as “good” or “bad” as molecules possess numerous functions that at one point in time may be beneficial (e.g., TNF-mediated neutrophil activation to kill a pathogen) or harmful (e.g., TNF-mediated lymphocyte apoptosis leading to immune paralysis). Second, as reviewed above, there is increasing evidence that both pro- and anti-inflammatory mediators can be equally dysregulated and imbalanced in the many critical septic patients—often simultaneously. Thus, it is imperative that what is designed to be a “functional” response to pathogen invasion of the host can be effectively regulated so that immune homeostasis can be preserved. The concept of utilizing CRRT modalities to not simply remove mediators from the circulation, but rather reduce and balance them to achieve homeostasis has been described as the so-called, “peak-concentration” hypothesis espoused by Ronco and colleagues [85]. The basis of this hypothesis is the concept that reducing the peaks of circulating mediators through use of extracorporeal filtration methodologies can substantially reduce their pathophysiologic impact. In testing this hypothesis over the past decade, several considerations in terms of timing, dosing and modality have been considered, but identifying distinct, effective approaches has proved challenging.

With use of CRRT, three mechanisms exist for mediator removal and consequent “blood purification”: convection, diffusion, and/or adsorption [86]. As a result, clinician-investigators have attempted to maximize these various approaches using CVVH with or without dialysis in order to determine if they could improve clinical outcomes in sepsis. Given that a higher dosing of CVVH should be associated with greater convective clearance of mediators, studies prospectively examined this treatment effect. Early promising results in adults were obtained by Ronco et al. who tested CVVH doses of 20, 35 and 45 ml/kg/h in a 425 adult ICU cohort with AKI, and observed improved survival in the higher-dose groups (35 and 45 ml/kg/h) as compared to the 20 ml/kg/h group [87]. However, because only ∼15 % of those enrolled had sepsis it was not possible to delineate a specific potential benefit in this minority of the cohort. Thus, in a subsequent study of over 200 AKI patients with over half (60 %) admitted with sepsis, higher-dose (42 ml/kg/h) versus “standard” CVVHD (24 ml/kg/h) showed improved survival giving credence to the possibility that higher “dosing” of hemofiltration—possibly due to greater mediator clearance—may have led to improved outcomes [88]. However, two recent trials that enrolled a substantially higher proportion of adults with sepsis (>60 %) did not support this conclusion [89, 90]. These observations in adults may not be relevant for pediatricians since with infant and child sizes with consequent estimated blood volume and achievable blood flow rates, even higher “dosing” of CVVHD can be delivered in children such that the studies examining even higher volume hemofiltration may be more directly relevant to determining the benefit of such an approach. In fact, a small trial of 33 adult patients with septic shock randomized to either 35 ml/kg/h versus 100 ml/kg/h over a 6-h period, demonstrated significantly lower circulating IL-6 levels consistent with the ‘peak concentration’ hypothesis; however, the study was too small to determine the influence on mortality [91]. Unfortunately, though the peak concentration hypothesis seems logical, very few studies in pediatric patients with sepsis-induced AKI have examined the impact of high-volume hemofiltration in sepsis-induced AKI.

Because a beneficial effect of high-volume hemofiltration has not been definitively demonstrated, investigators have challenged the premise that current membranes that possess kilodalton sieving cutoffs >30 kDa should effectively eliminate such mediators via the convective route to return the host to immunologic homeostasis. Instead, three alternative approaches to augmenting mediator clearance have been espoused: (1) increasing the sieving coefficient by using higher cutoff membranes (e.g., >60 kDa); (2) leveraging adsorption capacity of membranes; and (3) using membranes that possess mediator-specific adsorption capacity (e.g., polymethylmethacrylate [PMMA] membranes). Unfortunately, each of these has only been tested in adult cohorts. A series of clinical studies have evaluated the use of high-cutoff (HCO) membranes in adults with septic shock, including one recent phase II trial that enrolled 30 patients and demonstrated a reduction of vasoactive infusion requirements that correlated to significant reductions in serum IL-6 levels using HCO as compared to equivalent dosing with CVVH [92, 93]. With regards to the use of membrane adsorption capacity, a very early study by De Vriese et al. suggested that the principal route of cytokine clearance in patients with sepsis was via adsorption to the filter membrane [94]. Taking this approach, the Bellomo group was able to demonstrate that frequent changes of CVVH filters (3 × 3 h each) as compared to a single filter (used over an equivalent 9 h) significantly reduced both pro- (IL-8) and anti-inflammatory (IL-10) cytokine levels and was associated with a faster improvement in hemodynamics as measured by vasoactive use [95]. Building upon the concept that adsorption could effectively remove circulating mediators to attenuate AKI and other sepsis-related organ dysfunction, researchers have combined CVVHD with an endotoxin-adsorbing PMMA membrane to reduce circulating cytokines in the hopes of attenuating organ dysfunction [96]. It is highly anticipated that the completion of a number of either on-going or recently completed trials comparing these various strategies to traditional methodologies will provide clinicians with optimal strategies for managing sepsis-induced AKI—as well as other end-organ dysfunction. These studies are almost exclusively being performed in adults with sepsis who not only have different sepsis-induced hemodynamic profiles and development-dependent gene expression and immune function, but also have different physiologic responses to extracorporeal CRRT based on size, blood flow rates and consequently on dosing. Thus, it behooves the pediatric nephrology community to partner with intensive care and other colleagues to collaborate in an effort to examine similar therapeutic questions in the pediatric sepsis cohort.

## Conclusions

Our understanding of the pathophysiology of sepsis has grown substantially over the past two decades. While the immune response to pathogen invasion is a critical function that enables the host to contain and eradicate infections, dysregulation of both the pro- and anti-inflammatory components of this response substantially alter host physiology with consequent effects on end-organ function. The kidney is one of the principal targets of this pathophysiology and preserving its function in the setting of sepsis remains a fundamental challenge that necessitates both further understanding of the pathophysiologic mechanisms and the renal responses to therapeutic interventions. Furthermore, AKI (as well as ESRD) appears to place the host at greater risk for developing infectious complications so that attention must be paid to measures directed at preventing triggers of infections/sepsis in this at-risk cohort. In the event that AKI progresses to the point of needing RRT, further understanding of the ability of extracorporeal approaches to modulate the immune system during sepsis is necessary. Thus, several areas of on-going inquiry must be pursued to further advance our ability to successfully avoid or manage sepsis-induced AKI, as has been suggested by others, including: optimizing animal models that better mimic human pathology, determining the role of innate and adaptive immunity in sepsis-induced AKI and its potential modulation by extracorporeal approaches, identifying better scoring methodologies and biomarkers to identify AKI earlier, and examining the impact of the various therapeutic interventions directed at reducing circulating mediators that are responsible for end-organ injury that includes, but is not limited to, the kidney [97]. It is time for a multi-disciplinary approach to advancing both our pathophysiologic understanding of the mechanisms as well as the optimal therapeutic strategies that can attenuate the devastating consequences of sepsis-induced AKI in pediatrics.
